# OP81 Experience Matters: A Discrete Choice Experiment Exploring Patient Preferences For Heart Valve Procedures

**DOI:** 10.1017/S0266462324001387

**Published:** 2025-01-07

**Authors:** Simon Fifer, Brittany Keen, Polo Guilbert‐Wright, Dale Murdoch

## Abstract

**Introduction:**

Treatment for heart valve diseases (HVD) typically involves surgery, but less invasive procedures are becoming more common. Although the two procedures have similar outcomes, the risk–benefit profiles differ, indicating patients should be included in treatment decisions so they align with their values/preferences. This study aimed to determine patients’ preferences for HVD procedures, and the relative importance of treatment attributes.

**Methods:**

An online survey with discrete choice experiment (DCE) was disseminated to patients with aortic stenosis, mitral valve regurgitation, and tricuspid valve regurgitation. Participants were presented with several choice sets, each comprising two hypothetical treatment procedures (labeled “invasive procedure” and “minimally invasive procedure”) as well as an opt-out. DCE attributes were selected based on a literature review, qualitative interviews with patients and specialist doctors, and steering committee consultation (patients, patient organization representatives, and cardiac physicians). Responses were collected via healthcare recruiters, online panels, and patient organizations. DCE data from 143 Australian patients was analyzed using a mixed multinomial logit (MMNL) model.

**Results:**

Results indicate an “experience effect” whereby patients preferred the same type of treatment they had undergone previously. For example, patients who had undergone a transcatheter procedure were more likely to choose the minimally invasive procedure in the experiment and vice versa for those who had undergone invasive procedures like open-heart surgery. Patients were willing to switch procedures based on its risk–benefit profile, and most patients preferred the minimally invasive procedure when it reflected the profile of transcatheter aortic valve replacement (TAVI), even if they had previous invasive procedures experience. Key attributes driving choice were valve durability and regaining independence.

**Conclusions:**

There is a great deal of heterogeneity in HVD patient preferences, even when treatment outcomes appear similar. Patients preferred a minimally invasive procedure over an invasive procedure, irrespective of prior treatment experience with valve durability and independence driving choice. These results can inform healthcare decision-makers about what features of HVD procedures patients value most, taking into consideration patients’ prior experiences.

